# Irritable bowel syndrome and erectile dysfunction in medical students at a Peruvian university: an analytical cross-sectional analysis

**DOI:** 10.1093/sexmed/qfae021

**Published:** 2024-05-07

**Authors:** Mario J Valladares-Garrido, Luis E Zapata-Castro, Pedro P Quiroga-Castañeda, Iván Berrios-Villegas, Víctor J Vera-Ponce, Darwin A León-Figueroa, César J Pereira-Victorio, Danai Valladares-Garrido

**Affiliations:** Escuela de Medicina, Universidad Cesar Vallejo, Piura, 20001, Peru; Oficina de Epidemiología, Hospital Regional Lambayeque, Chiclayo, 14012, Peru; Faculty of Medicine, Universidad Nacional de Piura, Piura, 200104, Peru; Facultad de Medicina, Universidad de San Martín de Porres, Chiclayo, 14012, Peru; Facultad de Medicina, Universidad de San Martín de Porres, Chiclayo, 14012, Peru; Instituto de Investigación de Enfermedades Tropicales, Universidad Nacional Toribio Rodríguez de Mendoza de Amazonas, Chachapoyas, 010101, Peru; Universidad Tecnológica del Perú, Lima, 15046, Peru; Facultad de Medicina, Universidad de San Martín de Porres, Chiclayo, 14012, Peru; School of Medicine, Universidad Continental, Lima, 15046, Peru; Escuela de Medicina, Universidad Cesar Vallejo, Trujillo, 13001, Peru; Office of Occupational Health, Hospital Santa Rosa, Piura, 20008, Peru

**Keywords:** irritable bowel syndrome, erectile dysfunction, medical students, Peruvian

## Abstract

**Background:**

There is inconclusive evidence regarding the role of irritable bowel syndrome (IBS) in the development of erectile dysfunction (ED), especially among medical students due to high academic stress.

**Aim:**

To determine the association between IBS and ED in medical students from a Peruvian university in 2022.

**Methods:**

An analytical cross-sectional study was conducted with secondary data analysis on 133 medical students from a university in northern Peru during the 2021-II academic semester. The dependent variable was ED as measured with the 5-item International Index of Erectile Function, and the exposure variable was IBS as assessed with the Rome IV–Bristol questionnaire.

**Outcomes:**

The results were the prevalence rates of IBS and ED and the association of these variables.

**Results:**

Of the 133 medical students surveyed, the median age was 22 years (IQR, 19-24). The median score on the 5-item International Index of Erectile Function was 21 (IQR, 10-24). The prevalence of ED was 38.4% (95% CI, 30.05%-47.17%). Among the medical students 3% and 9% displayed moderate and severe ED, respectively, and 24.8%, 13.5%, and 24.1% showed moderate depressive, anxious, and severe symptoms. An overall 10.5% had IBS. Medical students with IBS had a 108% higher prevalence of ED than those without the syndrome (prevalence ratio, 2.08; 95% CI, 1.06-4.06). Other confounding variables were not significantly associated (*P* > .05).

**Clinical Implications:**

The results underline the importance of comprehensive sexual and mental health assessment, with an emphasis on the relationship between IBS and ED in medical students.

**Strengths and Limitations:**

Strengths include the use of validated and reliable instruments and rigorous biostatistical methods, and this is the first Peruvian investigation to explain the association between IBS and ED in medical students. Limitations include the cross-sectional design and nonprobability sampling, and there may be bias in applying the instruments.

**Conclusion:**

This study reveals a significant association between IBS and a higher prevalence of ED in these students.

## Introduction

Irritable bowel syndrome (IBS) is a functional gastrointestinal disorder characterized by clinical manifestations such as pain, abdominal distension, and alterations in defecation.^1^ The Rome IV criteria are considered the gold standard for diagnosis of IBS.^1^ IBS has a global prevalence of 3.8% in general populations according Rome IV criteria.^2^ Among medical students, this rate escalates to 30.9% to 36.3%.^3^^,^^4^ In Latin America, its prevalence ranges from 3% to 25%, with Peru reporting a rate of 21% for its general populace^3^^,^^5^ and 9.5% to 12.4% among medical students.^4^^,^^6^ The pivotal role of IBS is endothelial dysfunction resulting from chronic inflammation, predisposing individuals to vascular risks and consequently erectile dysfunction (ED) caused by impaired nitric oxide availability from inflammatory cytokines.^7^

In the context of chronic illnesses, sexual functions often become compromised, particularly in gastrointestinal disorders such as IBS, thereby revealing an association with ED.^8^ Defined as a persistent inability to maintain satisfactory sexual relations, ED has emerged as the most prevalent disorder in the general population, affecting 1% to 10% of young adults.^9^ Medical students, continually exposed to numerous stressors,^10^ warrant particular attention given their susceptibility to health-related quality-of-life issues.^11^

The pressures during medical training, financial strains, and poor dietary habits are among the primary concerns linked to mental health in medical students.^12^ These pressures are intensified by high academic workloads, strict attendance policies, and recurrent assessments, and students repeatedly cite these elements as major stress sources.^13^ Given these factors, there might be a shared pathophysiology between IBS and ED, grounded in endothelial dysfunction leading to associated systemic conditions such as cardiovascular disease.^8^

This correlation between IBS and the onset of ED in medical students remains an underexplored area. Primarily, there is a notable scarcity of firsthand data on ED incidence due to IBS in young adult university students, especially medical students, which hampers the comprehensive understanding of this relationship.^7^^,^^8^ Furthermore, some prior studies fall short in utilizing standardized diagnostic criteria, further muddling evaluations.^14^^,^^15^ The underlying mechanisms remain underresearched, emphasizing the need to pinpoint key associated factors. Also, certain previous findings are constrained by their sample sizes, making it challenging to extrapolate results to the broader student population. These are often descriptive and may not always employ specific association measures.^16^^,^^17^

Our research endeavors to address these limitations systematically, aiming to bridge the existing knowledge gap. As such, conducting this study could elucidate the association, laying the groundwork for tailored health strategies beneficial for medical students. It is crucial to emphasize that existing data pertaining to the association between IBS and ED are presently insufficient.

Consequently, the primary objective of our study is to ascertain the association between IBS and ED among medical students at a Peruvian university in 2022. Thus, our research question is as follows: Is there an association between the presence of IBS and the occurrence of ED in medical students at a Peruvian university?

## Methods

### Study design

This cross-sectional study is based on a secondary data analysis. The primary study evaluated factors associated with IBS in medical students from the Universidad San Martin de Porres–Northern Branch, located in the Lambayeque region, Peru, during the 2021–II semester.

### Population and sample

The primary study population consisted of 1325 medical students enrolled in their first to seventh year of studies during the 2021-II academic semester at the Universidad San Martin de Porres.

The primary study’s inclusion criteria were enrollment during the 2021-II academic semester and answering the IBS interest questionnaire. Students were excluded who did not provide informed consent, those reporting a diagnosis of IBS, and students with gastrointestinal pathologies or surgery within the last year.

A nonprobabilistic sampling was conducted. The estimated sample size for the primary study was 340 students, considering a population of 1325, a 5% precision, an expected prevalence of 37.3%,^18^ and a 95% confidence level. Additionally, 20% was added for potentially incomplete questionnaires. Eventually, 405 medical students were recruited for the primary study.

For the secondary data analysis study, only male students were included (*n* = 137). Those who did not correctly answer the ED questionnaire (5-item International Index of Erectile Function [IIEF-5], *n* = 4) were excluded.

### Instruments

#### ED questionnaire: IIEF-5

The IIEF-5 evaluates alterations in sexual phases such as desire, erection, orgasm, and ejaculation. Additionally, it assesses satisfaction in sexual relationships. It contains 5 items, each with responses on a 5-point Likert scale (range, 0–5). ED severity is scored from 5 to 25, with values between 22 and 25 (absent), 17 to 21 (mild ED), 12 to 16 (mild to moderate ED), 8 to 11 (moderate ED), and 5 to 7 (severe ED). A comparative study of the IIEF-5 and IIEF-15 yielded Cronbach’s alphas of 0.923 and 0.951, respectively.^19^ An adapted version for Colombian university students reported a Cronbach’s alpha of 0.91, sensitivity of 0.98, and specificity of 0.88, making it an optimal tool for diagnosing ED.^20^^,^^21^

#### Rome IV–Bristol questionnaire

A questionnaire was designed to diagnose patients with IBS, comprising 6 questions based on the new Rome IV criteria: 1 dichotomous question (yes or no), 1 image selection question (Bristol scale), 3 multiple-choice questions (always, 100%; almost always, 66%; sometimes, 33%; and never, 0%), and 1 question on frequencies. To diagnose, certain criteria must be met: Q42 (1 day a week) + Q46 (yes) + 2 of Q43, Q44, and Q45 with a minimum response of *sometimes*.^22^ Q48 was used to classify subtypes based on the Bristol visual scale. Previous studies reported that this questionnaire has a Cronbach’s alpha of 0.82 to 0.92, 62.7% sensitivity, and 97.1% specificity for identifying IBS in university students.^23^ This instrument demonstrates optimal psychometric properties, as indicated in a study encompassing 26 countries from various geographic regions, including Latin America. Adequate estimates were obtained in its exploratory factor analysis, supporting the international validity and reliability of the instrument.^24^

#### Depression, Anxiety, and Stress Scales–21

The Depression, Anxiety, and Stress Scales–21 consist of 21 items divided into 3 subscales (depression, anxiety, and stress). Responses are on a Likert scale ranging from 0 (“Did not describe me at all”) to 3 (“Yes, it happened a lot, or almost always”), with a maximum score of 21 for each subscale.^25^^,^^26^ Established cutoffs are provided for depression, anxiety, and stress levels. Previous reports on adult populations, including university students, indicate a Cronbach’s alpha of 0.91.^27^ For university students at health risk, the measure demonstrated specific sensitivity and specificity values for depression, anxiety, and stress.^28^ It has been validated in undergraduate students in Chile, demonstrating robust psychometric properties, including convergent validity, divergent validity, and reliability.^27^

#### Variables

The independent variable was IBS, clinically defined per the Roma IV criteria. The dependent variable was ED, operationally defined as a score <22 from the ED questionnaire (IIEF-5). Secondary independent variables included age, academic year, body mass index, frequent alcohol and tobacco consumption, regular physical activity, sleep quality, and mental health outcomes (depression, anxiety, stress).

### Study procedure

Initially, data collection forms were designed with the REDCap system. After obtaining approval from the university and ethics committee, we generated a self-administered survey link, contacting the study population through official social media groups of all academic years at the participating university. Participants took approximately 12 minutes on average to complete the online survey, with data immediately saved in the REDCap database. We then conducted quality control processes and exported the data to Stata version 17.0 (StataCorp LP).

### Statistical analysis

In the descriptive analysis, frequencies and percentages were presented for categorical variables. For numerical variables, such as age, the most appropriate central tendency and dispersion measure were reported after the assumption of a normal distribution was assessed.

In the bivariate analysis, associations were explored between IBS and ED, as well as potential confounding variables. For categorical independent variables, the chi-square test was used. For the numerical variable “age,” the Mann-Whitney *U* test was used after normal distribution assumptions were assessed. A 5% significance level was used.

In simple regression analysis, the association between IBS and ED, as well as secondary independent variables (confounding), was assessed via generalized linear models by employing the Poisson family, log link function, and robust variance. For multiple regression, the association of interest was controlled for confounding variables. We evaluated collinearity among the variables of interest and estimated prevalence ratios (PRs) and 95% CIs. The academic year variable was used as a cluster. Stata software was used for statistical analysis.

### Ethical considerations

The research was reviewed and approved by the Ethics and Research Committee of the Universidad San Martin de Porres (official document 040-2022). We sought informed consent before medical students’ participation and ensured data confidentiality through anonymous questionnaires and an anonymized database.

## Results

### Socioeducational characteristics of medical students

About 14.3% and 7.5% of students reported frequent alcohol and tobacco consumption, respectively. Just over one-third engaged in regular physical activity (38%). Depression, anxiety, and stress symptoms of a moderate nature were reported by 24.8%, 13.5%, and 24.1%. IBS was identified in 10.5% of the participants. The remaining results are presented in Table 1.

**Table 1:** Socioeducational characteristics of human medicine students (*N* = 133).

**Characteristic**	**No. (%)**
Age, y, median (IQR)	22 (19-24)
Academic year^a^	
1	15 (11.6)
2	11 (8.5)
3	25 (19.4)
4	16 (12.4)
5	17 (13.2)
6	16 (12.4)
7	29 (22.5)
Do you frequently consume alcohol?	
No	114 (85.7)
Yes	19 (14.3)
Do you frequently consume tobacco?	
No	123 (92.5)
Yes	10 (7.5)
Do you exercise regularly?^a^	
Yes	80 (62.0)
No	49 (38.0)
Level of depression	
Normal	56 (42.1)
Mild	25 (18.8)
Moderate	33 (24.8)
Severe	9 (6.8)
Extremely severe	10 (7.5)
Level of anxiety	
Normal	59 (44.4)
Mild	29 (21.8)
Moderate	18 (13.5)
Severe	6 (4.5)
Extremely severe	21 (15.8)
Stress level	
Normal	66 (49.6)
Mild	18 (13.5)
Moderate	32 (24.1)
Severe	15 (11.3)
Extremely severe	2 (1.5)
Hours of sleep^a^	
<8 h/d	100 (77.5)
≥8 h/d	29 (22.5)
Body mass index^a^	
Normal	70 (55.1)
Overweight	49 (38.6)
Obesity	8 (6.3)
Irritable bowel syndrome	
No	119 (89.5)
Yes	14 (10.5)
Erectile dysfunction	
No	82 (61.7)
Mild	20 (15.0)
Mild-moderate	15 (11.3)
Moderate	4 (3.0)
Severe	12 (9.0)

^a^Some variables do not add to 133 due to missing data

### ED questionnaire: IIEF-5

We found that 38.4% of the medical students exhibited some degree of ED (95% CI, 30.05%-47.17%). Furthermore, 3% and 9% of the medical students experienced moderate and severe ED, respectively (Table 1).

### IBS and ED in bivariate analysis

Our findings show that medical students with IBS had a 37% higher frequency of ED than those without the syndrome (71.4% vs 34.5%; *P* = .007). No statistical significance was observed for the association between ED and the other confounding variables (*P* > .05; Table 2).

**Table 2:** Irritable bowel syndrome and erectile dysfunction in medical students: bivariate analysis.

	**Erectile dysfunction, No. (%)**		
**Variable**	**No (n = 82)**	**Yes (n = 51)**	***P* value** ^a^
Age, y, median (IQR)	22 (19-24)	21 (19-26)	.678^b^
Academic year			.473
1	9 (60.0)	6 (4.0)	
2	8 (72.7)	3 (27.3)	
3	16 (64.0)	9 (36.0)	
4	7 (43.8)	9 (56.3)	
5	10 (58.8)	7 (41.2)	
6	13 (81.3)	3 (18.8)	
7	19 (65.5)	10 (34.5)	
Do you frequently consume alcohol?			.716
No	71 (62.3)	43 (37.7)	
Yes	11 (57.9)	8 (42.1)	
Do you frequently consume tobacco?			.181^c^
No	78 (63.4)	45 (36.6)	
Yes	4 (40.0)	6 (6.0)	
Do you exercise regularly?			.418
No	53 (66.3)	27 (33.8)	
Yes	29 (59.2)	20 (4.8)	
Level of depression			.372
No	37 (66.1)	19 (33.9)	
Yes	45 (58.4)	32 (41.6)	
Level of anxiety			.097
No	41 (69.5)	18 (3.5)	
Yes	41 (55.4)	33 (44.6)	
Stress level			.912
No	41 (62.1)	25 (37.9)	
Yes	41 (61.2)	26 (38.8)	
Hours of sleep			.804
<8 h/d	63 (63.0)	37 (37.0)	
≥8 h/d	19 (65.5)	10 (34.5)	
Body mass index			.384^c^
Normal	43 (61.4)	27 (38.6)	
Overweight	31 (63.3)	18 (36.7)	
Obesity	7 (87.5)	1 (12.5)	
Irritable bowel syndrome			**.007** ^d^
No	78 (65.6)	41 (34.5)	
Yes	4 (28.6)	10 (71.4)	

^a^ *P* value of categorical variables calculated with the chi-square test.

^b^ *P* value of categorical numerical variables calculated with the Mann-Whitney *U* test.

^c^ *P* value of categorical variables calculated with Fisher’s exact test.

^d^ *P* < .05.

### IBS and ED in simple and multiple regression analysis

Having IBS increased the prevalence of ED among medical students by 122% (PR, 2.22; 95% CI, 1.43-3.46). No confounding variables associated with ED were observed (Table 3).

**Table 3:** Irritable bowel syndrome and erectile dysfunction: simple and multiple regression analysis.

	**Erectile dysfunction**					
	**Simple regression**			**Multiple regression**		
**Characteristic**	**PR**	**95% CI**	***P* value** ^a^	**PR**	**95% CI**	***P* value** ^a^
Age, y	1.00	0.92-1.08	.998	1.00	0.94-1.08	.806
Do you frequently consume alcohol?						
No	1 [Ref]			1 [Ref]		
Yes	0.71	0.32-1.57	.395	0.70	0.33-1.46	.340
Do you frequently consume tobacco?						
No	1 [Ref]			1 [Ref]		
Yes	0.91	0.31-2.64	.864	0.97	0.52-1.78	.909
Do you exercise regularly?						
Yes	1 [Ref]			1 [Ref]		
No	1.21	0.98-1.50	.080	1.15	0.89-1.48	.294
Level of depression						
No	1 [Ref]			1 [Ref]		
Yes	1.27	0.73-2.22	.401	0.99	0.49-1.98	.294
Level of anxiety						
No	1 [Ref]			1 [Ref]		
Yes	1.53	0.82-2.86	.179	1.57	0.64-3.88	.327
Stress level						
No	1 [Ref]			1 [Ref]		
Yes	1.03	0.69-1.54	.896	0.76	0.42-1.40	.382
Hours of sleep						
<8 h/d	1 [Ref]			1 [Ref]		
≥8 h/d	0.93	0.52-1.68	.815	1.00	0.51-1.93	.989
Body mass index						
Normal	1 [Ref]			1 [Ref]		
Overweight	0.95	0.52-1.74	.874	0.86	0.43-1.74	.680
Obesity	0.32	0.06-1.81	.199	0.35	0.06-1.94	.229
Irritable bowel syndrome						
No	1 [Ref]			1 [Ref]		
Yes	2.22	1.43-3.46	**<.001** ^b^	2.08	1.06-4.06	**.032** ^b^

Abbreviations: PR, prevalence ratio; Ref, reference.

^a^ *P* values obtained with generalized linear models, Poisson family, log link function, robust variance, and cluster by academic year.

^b^ *P* < .05.

In multiple regression analysis, findings from the simple regression persisted in magnitude and direction. We found that medical students with IBS had a 108% higher prevalence of ED than those without the syndrome (PR, 2.08; 95% CI, 1.06-4.06). No other confounding variables were significantly associated (*P* > .05; Table 3).

## Discussion

### Prevalence of ED

We found that 38.4% of the students had ED. Of these, 15% and 11.3% presented with mild and mild-moderate ED, respectively. In addition, 3% had moderate ED and 9% had severe ED. These results closely align with the findings of Mialon et al in a study conducted among individuals aged 18 to 25 years in Switzerland, where the prevalence of ED was 29.9%.^29^ Mild to moderate cases were more frequent, accounting for 25.5% as determined by the IIEF-5.^29^ However, this differs from the findings of Moreira et al, who observed a 46.2% prevalence of ED among young adults in Brazil, including individuals with varying levels of education. In their study, 31.5% had mild ED, 12.1% moderate, and 2.6% severe.^30^ It is important to note that the reference stratifies the sample by level of education rather than providing a specific analysis of the population of university students. These results differ from the findings of Gutierrez-Velarde et al in a study conducted among students at a university in Lima, Peru, where the prevalence of ED was 54.6%.^31^ Within this, 43.3% of cases were categorized as mild and 11.3% as mild to moderate, according to the IIEF-5.^31^ Our findings are consistent with those of Santibáñez et al, who reported a 26.2% prevalence of mild-moderate ED among young Peruvian adults, even though data in our context are rather limited.^32^ These high rates might be explained by the fact that young adults often struggle to discuss their sexual health during clinical visits.^33^ In fact, 68% of medical students are hesitant to discuss ED due to feelings of embarrassment with a health care professional, and 71% believe that doctors would dismiss their concerns.^34^ Against this backdrop, it has been shown that medical students often face immense workloads and significant academic demands. They are also exposed to stressful and emotionally intense situations, which can negatively affect their physical and mental health. Consequently, chronic anxiety and stress can influence the production of sex hormones and the vascular-neural function necessary for erection, making them more susceptible to ED.^35–37^ Moreover, a UK medical education report determined that ED is one of the least-taught topics in medical schools, with 43.8% coverage, potentially explaining gaps in knowledge, skills, and attitudes regarding its prevention and proper management.^38^ Coupled with the fact that ED is correlated with reduced physical-emotional satisfaction and can be linked to poor quality of life, depression, and lack of adherence to specific treatments,^33^ there is an urgent need to implement health guidance and monitoring programs in vital environments such as universities. Such initiatives could significantly influence medical training and education. It is important to note that variations in reported prevalence rates across studies may also be attributed to differences in sampling methods. The use of random^30^^,^^32^ or nonrandom^39^ sampling in similar studies can introduce variability in the composition of study populations, influencing the observed rates of ED.

### Association between IBS and ED

Students with IBS showed a higher prevalence of ED, consistent with the findings of Chao et al, who determined in a cohort study that the incidence rate of ED was 2.92 times higher in patients in Taiwan with IBS as compared with those without IBS. Additionally, individuals with IBS had an instantaneous risk 2.58 times higher of experiencing ED (adjusted hazard ratio, 2.58; 95% CI, 2.24-2.98).^8^ This is consistent with the findings of Hsu et al in another cohort study, who reported that having IBS increased the instantaneous risk of developing organic ED by 2.12 times (hazard ratio, 2.12; 95% CI, 1.80-2.50) and psychogenic ED by 2.38 times (hazard ratio, 2.38; 95% CI, 1.47-3.85).^7^

This observed association may be due to the fact that individuals with IBS often experience diminished quality of life resulting from the disease, which commonly results in a significant mental and emotional impact.^40^^,^^41^ Adding to this, the high levels of stress faced by medical students due to the rigorous demands of their academic pursuits could increase the risk of ED as it might affect sexual function.^42^ Moreover, it has been demonstrated that patients with IBS have alterations in gut hormonal regulation, including serotonin, motilin, and peptide YY. These hormones are involved in regulating sexual function.^43^ Another potential explanation might be vascular factors since ED can be related to endothelial dysfunction, and IBS can affect endothelial function due to chronic inflammation and gut dysbiosis, potentially contributing to ED.^7^ Indeed, evidence suggests that those with IBS are almost 3 times more likely to experience ED than those without the condition.^8^^,^^35^ These findings mainly come from older studies since there is a lack of literature focusing on medical students in training.^44^^,^^45^ Nevertheless, they highlight the pressing need to conduct updated research to bridge the knowledge gap for this age group, which faces substantial emotional and academic burdens in the realm of medical education.

### Mental health and ED

We did not identify that the presence of depressive, anxious, and stress symptoms was associated with ED in male students. This is consistent with the literature, as healthy sexuality and relationships may act as a protective factor against the aforementioned symptoms in medical students.^46^ However, reverse causality could also exist; that is, ED can cause mental health symptoms and vice versa.^47^ In a systematic review, Liu et al estimated that men with depression had a 2.92-times higher probability of experiencing ED (odds ratio, 2.92; 95% CI, 2.37-3.60).^48^ In Chinese men aged 22 to 50 years, Zhang et al found a higher proportion of ED in those with depressive and anxious symptoms (*P* = .002).^49^ Yang et al identified a correlation between ED and depressive symptoms (*r*^2^ = –0.653) as well as anxious symptoms (*r*^2^ = –0.607) in Chinese men.^50^ Likewise, a systematic review including studies conducted in Latin America reported that young adults with mental health disorders had a median prevalence of 20% for ED (IQR, 20%).^51^ The negative association that we report might be explained by 2 facts. First, there may be high resilience in the studied population to handle stress and strong emotional burdens during the challenging journey of becoming a doctor, even though it has been reported that the psychogenic etiologies of ED mainly involve the development of depression and anxiety.^37^ Second, the relationship between mental health and ED might be bidirectional,^51^ representing a valuable opportunity to explore and understand the main factors influencing the psychological response of medical students with ED.

### Relevance of findings in public health

In our view, documenting the association between IBS and ED in medical students is highly relevant. First, it would enhance the understanding and treatment of both clinical pictures as they seem to have a bidirectional relationship.^51^ Second, having firsthand data will greatly assist in presenting solid evidence to identify “silent” comorbidities for students in training. Ultimately, our study will serve as a niche for future research related to this topic, and it especially aims to increase the sensitivity of the affected population so that more timely strategies can be implemented for promotion, prevention, and control.

Additionally, it was demonstrated that among the main predictive factors in patients with IBS, drug abuse, lifestyle, body mass index, perceived physical fitness, depression, and perceived mental health deterioration were associated with the development and persistence of ED in university students.^52^ This reflects that health professionals should also adequately assess the accompanying psychological symptoms that contribute to the sexual health of patients with IBS,^37^ as it has been shown that in the young adult population, the most frequent cause of ED tends to have a psychogenic basis.^53^ Similarly, this is an aspect that should be managed holistically in different study centers, such as universities, as it would represent a public health strategy ensuring a comprehensive assessment for students, especially for those training to be doctors, who will be future leaders of the Peruvian health system.

### Limitations and strengths

The study has certain limitations. First, the cross-sectional design does not allow for the inference of causality. Second, a notable limitation pertains to our sampling strategy. It is crucial to emphasize that random sampling was not conducted, introducing the potential for selection bias. This means that the generalization of our findings to the broader population of medical students may be limited. Third, there may be biases inherent to the instruments used, such as social desirability bias, which occurs when study participants provide responses that they consider socially desirable instead of honest and accurate; as such, the data might not be representative of the actual situation. There could also be an underreporting bias, as some respondents might not provide valuable information because they find it shameful, stigmatized, or socially unacceptable. These biases might reduce the accuracy of the responses; however, to address these, we implemented instruments that provide detailed information with the specific temporality of the symptoms presented in both clinical pictures. Fourth, there might be a measurement bias since it was not possible to measure other variables potentially associated with ED, such as cardiovascular disease (diabetes, obesity), tobacco, alcohol, sleep quality, physical exercise, and drug consumption (antidepressants, antihistamines, diuretics, and antihypertensives).^54^^,^^55^ Additionally, we did not measure variables related to the impact of the COVID-19 pandemic, including a history of COVID-19 infection and specific psychosocial stressors, such as having a family member with COVID-19, experiencing the loss of a family member due to COVID-19, feelings of uncertainty, and fear of COVID-19.^56^ Nonetheless, this study has the following strengths. To our knowledge, this is the first Peruvian research that attempts to explain the association between IBS and ED in medical students, a scenario scarcely developed in our country and whose data are urgently needed to implement appropriate and culturally adapted intervention strategies. Furthermore, rigorous biostatistical methods have been used to explain each of the previously reported results, and the variables of interest have been measured by valid and reliable instruments with appropriate psychometric properties.^19–21^^,^^23^^,^^28^ Ultimately, this research significantly contributes to evidence generation, as the relationship between IBS and ED is not yet fully understood and more research is needed to establish a potential causal relationship.

## Conclusions

We found that medical students diagnosed with IBS exhibited a higher prevalence of ED. The significant relationship between IBS and ED underscores the need to address these issues holistically, considering physical and psychological factors. Additionally, our findings emphasize the importance of medical education in sexual and mental health and the implementation of promotion and prevention strategies in academic settings. Understanding the bidirectional relationship between IBS and ED is essential to provide effective medical care and improve the quality of life of medical students. These results also have broader implications in terms of public health, highlighting the importance of comprehensive care programs in educational institutions. These findings are relevant in the clinical and medical training spheres, and it is hoped that they will contribute to improving the health of medical students and ultimately the general population.

## Supplementary Material

STROBE-checklist-v4-cross-sectional_qfae021
